# Correlation analysis of epicardial adipose tissue volume quantified by computed tomography images and coronary heart disease under optimized reconstruction algorithm

**DOI:** 10.12669/pjms.37.6-WIT.4882

**Published:** 2021

**Authors:** Zhenwei Miao, Hongyan Yang, Bofen Liu, Wengui Li

**Affiliations:** 1 Zhenwei Miao, Master of Medicine. Department of Radiology, Tianjin Baodi Hospital, Tianjin City 301800, China; 2 Hongyan Yang, Bachelor’s Degrees. Department of Nursing, Tianjin Baodi Hospital, Tianjin City 301800, China; 3 Bofen Liu, Bachelor’s Degrees. Department of Nursing, Tianjin Baodi Hospital, Tianjin City 301800, China; 4 Wengui Li, Bachelor’s Degrees. Department of Radiology, Tianjin Baodi Hospital, Tianjin City 301800, China

**Keywords:** Ordered subset expectation maximization reconstruction algorithm, Coronary heart disease, Computed tomography, Epicardial adipose tissue, Gensini scoring

## Abstract

**Objectives:**

This paper was aimed to explore the adoption value of low-dose computed tomography (CT) imaging based on optimized ordered subset expectation maximization (OSEM) reconstruction algorithm in the correlation analysis between epicardial adipose tissue (EAT) volume and coronary heart disease (CHD).

**Methods:**

A total of 110 patients with CHD were selected for CT angiography (CTA) and coronary arteriography (CAG) examinations from October 2017 to October 2019. The predictive value of EAT for CHD was analyzed via receiver operating characteristic (ROC) curve.

**Results:**

The results showed that the iteration time and error of the improved OSEM reconstruction algorithm were better than that of MLEM algorithm under the same number of iterations. Age, smoking, hypertension, diabetes, and EAT in control group were obviously lower in contrast to those in CHD group (P<0.05). EAT in control group was (124.50±26.72) mL, and EAT in the CHD group was (159.41±38.51) mL. EAT (B=0.023, P=0.003) was an independent risk factor for CHD, which was suggested by Multiple linear regression analysis. Moreover, EAT was a risk factor for CHD, and was positively correlated with the degree and NSCV.

**Conclusion:**

The optimized OSEM algorithm was used to improve the reconstruction quality of low-dose CT images and used in quantitative measurement of epicardial fat volume. Results showed EAT was an independent risk factor for CHD, and was positively correlated with the number of coronary lesions and Gensini score. It was of great value for the prediction of CHD.

## INTRODUCTION

Many studies have revealed that EAT is closely related to the occurrence of coronary atherosclerosis, and may be a new risk factor for CHD.^1^-^3^ The measurements of EAT include ultrasound^4^-^6^, MRI^7^ and CT.^8^ CT imaging technology is currently the preferred method for measuring EAT in clinics, and it can achieve accurate quantitative measurement of the total volume of EAT. Therefore, using CT technology to explore the volume changes of the EAT in CHD patients is of great significance for the early prevention of CHD and the subsequent effective treatment.

Therefore, the optimized OSEM algorithm was applied in low-dose CT image reconstruction for CHD patients to obtain high-quality images. Quantitative measuring of the volume of epicardial fat in patients with CHD was performed, and its correlation with the incidence of CHD was further explored, to provide accurate imaging basis for early diagnosis of CHD.

## METHODS

One hundred and ten patients with CHD who were examined by CTA and CAG in our hospital from October 2017 to October 2019 were selected. Patients with CAG stenosis no less than 50% were classified as CHD group, and the rest as control group. Among them, there were 42 patients (28 males) in control group with an average age of 59.85±7.67 years. There were 68 patients (42 males) in the CHD group with an average age of 61.67±6.28 years.

### Inclusion criteria

I, patients were 30-84 years old; II, patients received CTA and CAG joint inspection; III, the image quality was favorable.

### Exclusion criteria

I, patients with coronary artery bypass graft; II, patients with coronary artery stenting; III, patients with congenital heart disease; IV, poor image quality; V, patients with atrial fibrillation and arrhythmia; VI, patients with cardiomyopathy or valvular disease. This experiment had been approved by the ethics committee of the hospital, and the patients involved in the study had known and agreed with it.

### ETA volumetric measurement

Two experienced physicians were selected to conduct ETA measurement using Vitrea post-processing workstation related software, and the results were averaged. The fat threshold range was set from 150 to -50Hu. Firstly, the fat tissue was extracted, mainly from the myocardium and the visceral pericardium. Then, measurements were made from the beginning of the left pulmonary artery to the apex of the left ventricle.

The detection was completed by two senior interventional cardiologists. Left and right coronary arteriography were performed by conventional body position imaging, and heparin was used for anticoagulation during the operation. Coronary angiography referred to diagnosing and analyzing the location, severity, and NSCV of coronary artery stenosis. The left main coronary artery was classified as two-vessel lesion, while other vascular lesions were classified into single-vessel, double-vessel, and multiple-vessel lesions according to the NSCV.

The Gensini scoring system was used to evaluate the degree of vascular lesions [9]. The stenosis coefficient value was determined, the diameter of blood vessel was “0-25%, 51%-75%, 76%-90%, 91-99%, and 100%”, which counted 1, 2, 4, 8, 16, and 32 points, respectively. The lesion site coefficient value was determined. The coefficient of left main coronary artery was 5. The coefficients of the proximal left anterior descending and the proximal left rotatory branch were both 2.5. The coefficient of the middle part of left anterior descending branch was 1.5. The coefficients of the distal left anterior descending branch, the distal left anterior branch, the obtuse margin branch, the right coronary artery (proximal, middle, and distal), and the posterior descending branch were all 1. The coefficient of the second diagonal branch was 0.5. The value of lesion site coefficient × stenosis coefficient value was the total score.

OSEM is an accelerated iterative algorithm. The main principle is sorting the projection data first, and then dividing it into L subsets {*S*_1_,*S*_2_,*S*_3_,...,*S_L_*}. In each sub-iteration process, all projection data of a certain subset are used to correct the image.^10^ Therefore, the image in each iteration is updated L times, which makes the convergence rate increase rapidly and the CT image reconstruction time becomes shorter. In addition, OSEM algorithm also has good anti-noise ability and high spatial resolution. The iteration equation is as follows.

In the above equation, j is the pixel; i is the detector number; qij is the probability; k is the number of iterations; gkj is the image after k iterations; yi is the observation value; sm is the m-th subset; and 1≤m≤L.

The specific implementation of the OSEM algorithm is divided into steps such as initial value, subset projection of the probability matrix, residual calculation, correction factor calculation, image correction, and loop iteration.^11^ The calculation of the initial value is as follows.


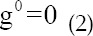


Projection of probability matrix on subset is as follows.


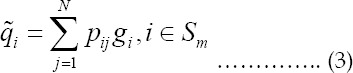


The ratio of the actual projection qi to the theoretical projection is the residual.


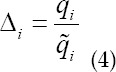


Correction factor calculation is as follows.


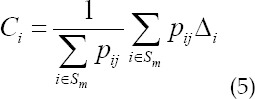


CT reconstruction image correction is as follows.


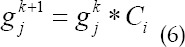


Optimizing of the partitioning of OSEM algorithm subsets is as follows. I, the number of projections was fully considered, so that all subsets obtained by the division have uniform projection angles, and the subsets should be balanced and symmetrical; II, in the subset sorting process, it should be ensured that the distance between the projection directions of the adjacent two subsets is the largest.

## RESULTS

The simulation data was a 256×256 sheep-Logan phantom, and each subset in the OSEM algorithm was set with 6 projection directions. When the projection interval was 1°, the iteration time was 223.56, and the error was 0.0465. The accuracy of the reconstructed image was very high. When the number of iterations increased, the error decreased and the imaging quality would be higher. The iteration time increased with the number of iterations.

The projection interval was set as 1° and the number of projection directions as 6. When the number of iterations was the same, the OSEM algorithm had shorter iteration time and smaller error. When the MLEM algorithm was iterated for 30 times, the error was at the same level as the OSEM algorithm after 1 iteration. It can be concluded that the calculation speed of OSEM algorithm was higher in contrast to that of MLEM algorithm, the reconstruction time was greatly shortened, and the imaging quality was better.

The results showed that middle part of anterior descending branch was about 80%, the proximal stenosis of the circumflex branch was between 70% and 75%, and the Gensini score was 36. With CHD as the dependent variable and gender, BMI, uric acid, smoking, hypertension, diabetes, and EAT as the independent variables for Logistic multiple regression, EAT (B=0.023, P=0.003) was an independent risk factor for CHD (Table-I).

**Table-I:** Risk factors for CHD.

*Factor*	*Wals*	*B*	*SE*	*OR*	*95% CI*		*P*

					*Lower limit*	*Upper limit*	
Constant	0.005	0.167	2.654	1.176	-	-	0.864
EAT	8.365	0.023	0.006	1.017	1.003	1.038	0.003
Uric acid	0.768	0.007	0.018	0.996	0.975	1.001	0.354
Age	0.685	0.236	0.028	0.398	0.956	1.023	1.007
BMI	2.187	-0.114	0.073	0.142	0.745	1.037	0.879
Gender	0.032	-0.117	0.627	0.874	0.238	3.109	0.865
Smoking history	3.218	-1.105	0.542	0.321	0.087	1.006	0.718
Hypertension	3.265	-1.486	0.821	0.068	0.039	1.076	1.206
Diabetes	2.483	-0.802	0.486	0.102	0.154	1.208	0.432

Spearman correlation analysis suggested that EAT volume was positively correlated with Gensini score (r=0.327, P=0.002); EAT volume was positively correlated with the NSCV (r=0.416, P=0.000) (Table-II).

**Table-II:** Correlation of EAT with Gensini score and the NSCV.

	*Gensisi score*	*Number of stenosed coronary vessel*
r	0.327	0.416
P	0.002	0.000

**Fig.1:**
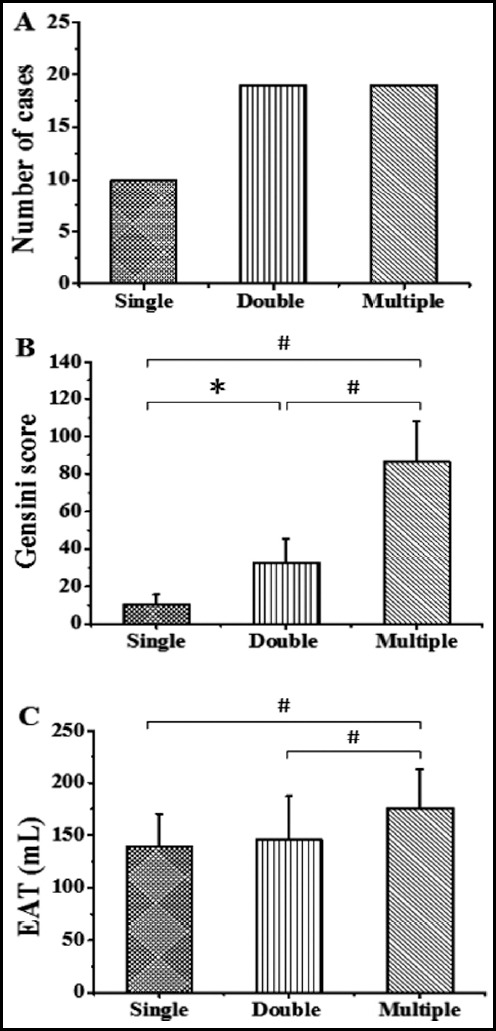
The relationship between NSCV and EAT and Gensini. (A: number of patients; B: Gensini score; C: EAT; * meant P<0.05; # meant P<0.01.)

Among CHD patients, there were 10, 19, and 19 cases of single-vessel, double-vessel, and multivessel disease (Fig.1A). The Gensini score of double-vessel disease was notably higher in contrast to that of single-vessel disease, P<0.05; the Gensini score of multivessel disease was extremely notably higher in contrast to those of double-vessel disease and single-vessel disease, P<0.01 (Fig.1B). The EAT value of multivessel disease was extremely notably higher in contrast to those of double-vessel disease and single-vessel disease, P<0.01 (Fig.1C).

## DISCUSSION

With the rapid development of medical imaging technology, X-ray, MRI, and CT have been widely used in the disease diagnosis, treatment, and efficacy evaluation. CT imaging is often used in the examination of chest and abdomen diseases. Chest CT examination has the advantages of clear structure, but the image is not very clear for soft tissue, and the radiation amount of CT imaging is much higher than that of X-ray.^12^,^13^ In order to reduce the amount of radiation to the human body during CT examination, low-dose CT is often used for clinical examination. However, as the intensity of CT scan decreases, the quality of the reconstructed image will be greatly reduced.^14^ Therefore, the reconstruction of low-dose CT images has become the focus of attention, and the use of iterative reconstruction algorithms to reconstruct low-dose CT images can obviously solve the reduced signal-to-noise ratio.^15^ The OSEM algorithm can divide the projection data into N different subsets through an iterative reconstruction process, and each subset is iterated.^16^,^17^ In this study, OSEM algorithm was used to reconstruct low-dose CT images. The results showed that, compared with MLEM algorithm, OSEM algorithm can significantly shorten the iteration time, and the image error after reconstruction was smaller. The MLEM algorithm has shortcomings, such as slow iteration speed, which is consistent with the results of this study.^18^

## CONCLUSIONS

The optimized OSEM algorithm was used to improve the reconstruction quality of low-dose CT images and used in quantitative measurement of epicardial fat volume. Results showed EAT was an independent risk factor for CHD, and was positively correlated with the number of coronary lesions and Gensini score. It was of great value for the prediction of CHD.

### Authors Contribution:

**ZM:** Conceived the study, literature review, data analysis, preparing the draft of paper.

**HY & BL:** Helped in design, data collection drafting & critical revision.

**WL:** Takes the responsibility and is accountable for all aspects of the work in ensuring that questions related to the accuracy or integrity of any part of the work are appropriately investigated and resolved.
